# DicePlot: a package for high-dimensional categorical data visualization

**DOI:** 10.1093/bioinformatics/btaf337

**Published:** 2025-06-17

**Authors:** Matthias Flotho, Philipp Flotho, Andreas Keller

**Affiliations:** Clinical Bioinformatics, Center for Bioinformatics, Saarland University, Saarland University Campus, 66123 Saarland, Germany; Helmholtz Institute for Pharmaceutical Research Saarland (HIPS), Saarland University, Saarland University Campus, 66123 Saarland, Germany; Clinical Bioinformatics, Center for Bioinformatics, Saarland University, Saarland University Campus, 66123 Saarland, Germany; Clinical Bioinformatics, Center for Bioinformatics, Saarland University, Saarland University Campus, 66123 Saarland, Germany; Helmholtz Institute for Pharmaceutical Research Saarland (HIPS), Saarland University, Saarland University Campus, 66123 Saarland, Germany

## Abstract

**Summary:**

Visualization of multidimensional, categorical data is a common challenge across scientific domains and, in particular, the life sciences. The goal is to create a comprehensive overview of the underlying data which enables one to assess multiple variables. One application where such visualizations are particularly useful is gene or pathway analysis, which involves checking for dysregulation in known biological mechanisms and functions across multiple conditions. Here, we propose a new visualization approach that encodes such data in an intuitive representation: DicePlots visualize up to four distinct categorical classes in a single view using elements resembling dice faces, whereas DominoPlots add an additional layer of information for binary comparison.

**Availability and implementation:**

The code is available as the *diceplot* R package and the *pydiceplot* on PyPI. All source code is available at https://github.com/maflot.

**Contact:**

The repo is managed actively and we encourage community contributions and requests.

## 1 Introduction

In biosciences, visualizing and representing data in an appealing and informative way is crucial for both data exploration and presentation. In single-cell RNA-sequencing studies, we face the problem that different conditions require visualizations allowing simultaneous assessment of multiple variables. In particular, visualizing pathway analyses across multiple cell types and conditions is challenging as we are often interested not only in the intersections across diverse conditions but also in the categories of each variable. There exist many established and excellent solutions for high-level data visualization. An important example are scatter plots of UMAP or PCA embeddings, which are used to visualize the structure of the underlying high-dimensional data, however, the interpretation of the dimensionality reduction method needs to be carefully considered (Chari and Pachter 2023). Venn diagrams (Venn 1880) are useful to highlight the intersections of different conditions. Moreover, approaches such as UpSet plots (Lex *et al.* 2014) are brilliant for visualizing the quantitative overlaps and represent a significant improvement over classical Venn diagrams when handling many groupings. Scalability and selection of the intersecting sections is not a trivial task. Finally, another well-established plot is the circle plot, which offers great options for visualizing and quantifying intersections between several groups (Gu *et al.* 2014). While all of those approaches are great for visualizing the quantitative overlap of groups, they lose all information about the elements of the intersections. For qualitative analyses, there exists numerous visualization techniques to examine categorical relationships and patterns within the data, whether across experimental conditions, cell types, or gene expression profiles. Important examples include bar charts for category comparison and scatter plots for distribution assessment. For quantitative comparisons, volcano plots effectively visualize both magnitude (fold change) and statistical significance (*P*-value) of differentially expressed genes between two conditions, while gene-gene scatter plots quantify expression correlation between specific genes. However, those approaches can again only visualize one aspect of the data. They are useful to visualize qualitative details of the data but are not useful for high-level overviews of the data. Here, we present Dice- and DominoPlots, intuitive data visualization that bridge high-level and low-level views of the data. The representation allows the display up to four different dimensions of the data in a DicePlot and double the number in a DominoPlot. This will serve as an addition to the established plotting approaches. These representations provide a comprehensive overview of shared attribute quantities while preserving important set information. We will show two examples of gene analysis that, display the functionality and complementary aspect of Dice-, and DominoPlots to common representations.

## 2 DicePlot

**Figure 1.**
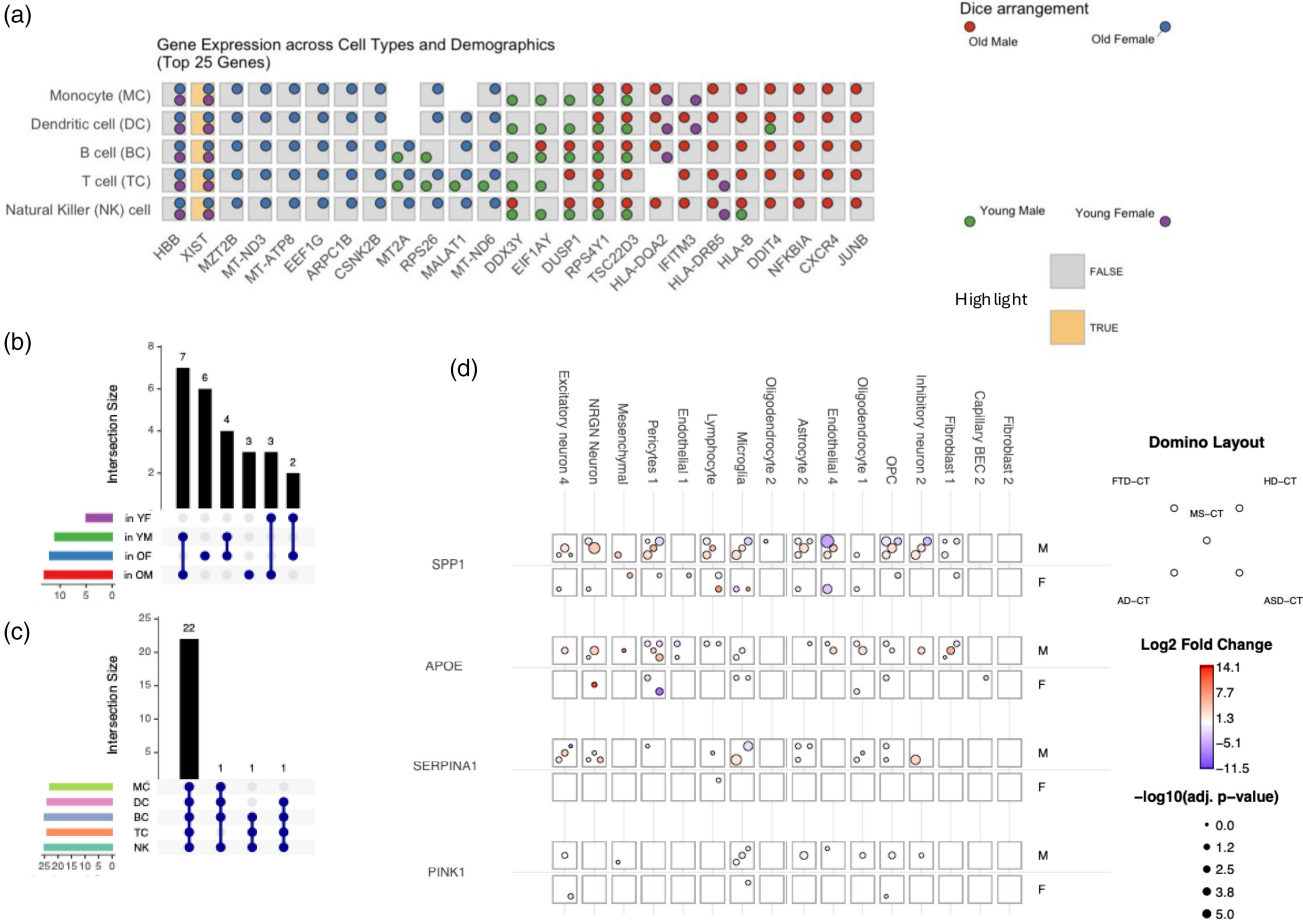
(a) Gene dysregulation across different PBMC populations young and old male and female individuals in both conditions visualized using a DicePlot from Fig. 3 in a study by Huang *et al.* (2021). On the right the legend shows the dot arrangement, and is clarifying which dice dot corresponds to which class, while the squares below show the class used for back-coloring the dice faces. (b and c) UpSet plots complementing the DicePlot representation, by directly highlighting the intersection distributions across different groupings, and cell types, while losing the information of the particular genes in the intersection. (d) A DominoPlot highlighting differentially expressed genes known to be linked to neurodegeneration across different cell types reported in the ZEBRA database (Flotho *et al.* 2024). Here, the color represents the Log2 fold changes, while the size corresponds to the negative log fold change. This representation enables in a single matrix to show the dysregulation for five differential expression tests.

Displaying highly dimensional categorical data is challenging without splitting the information into diverse subplots or losing categorical information. Here, we are able to display up to four distinct categorical variables in a single view. Our DicePlot visualization is inspired by the familiar pattern of dots on a six-sided die, where each dot position is fixed and recognizable. Each dot position on the die corresponds to a specific category of the variable being visualized, maintaining consistent positions across all dice in the visualization. The number of dots shown on each die directly represents the number of unique categories present in that particular data subset. We limited the design to six categories maximum to maintain visual clarity while avoiding excessive complexity, and to align with the intuitive six-sided die metaphor familiar to most viewers. The spatial arrangement follows traditional die face patterns, providing an immediate visual reference for quantity. When displaying three categories, we implemented an alternative triangular arrangement option to enhance clarity beyond the traditional diagonal. Variation information is encoded through the presence or absence of dots in specific positions—by comparing which dot positions are filled across multiple dice, viewers can quickly identify differences in category distribution. This approach allows for easy comparison across different subsets of data while maintaining consistent positional encoding. For emphasizing this we will take a look at an example dataset of gene dysregulation of diverse genes in different PBMC populations published by Huang *et al.* (2021) (Fig. 1a). The DicePlot is highlighting the different examined cell type populations on the y-axis, while the x-axis is showing the 25 genes with highest dysregulation. Huang *et al.* investigate this across four conditions: “Young males (YM),” “Old males (OM),” “Young females (YF),” “Old females (OF).” The objective is to visualize the genes specificity to certain cell types and sexes within a single view. In theory, the coloring of the different groups can be each either categorical or continuous, as the variation information is already encoded in the position of the dots. The positioning can be either set manually by defining a factor describing the order, or by clustering the categories. The DicePlot is able to directly pinpoint particular shared genes across conditions and cell types, it loses the broad overview of the intersections. For this we can use an UpSet plot to complement the DicePlot (Fig. 1b and c). The UpSet plot loses the information of the particular genes in the intersections, while maintaining a clean overview of the set sizes. Finally, the DicePlot can color the background of the dice according to another grouping variable to highlight certain boxes. In our example we chose to highlight XIST, a gene well known for being specific to female individuals and being dysregulated as expected only in the female group. In the context of pathway enrichment analysis, this could easily be used to highlight high-level groupings of the shown pathways.

## 3 DominoPlot

As a further addition and complement to volcano plots, we introduce the DominoPlot—a visualization designed to highlight key genes across multiple comparisons. While volcano plots become too overloaded when displaying genes from multiple comparisons in a single graph, or require separate plots for each comparison, the DominoPlot efficiently displays these changes across multiple conditions simultaneously. The DominoPlot trades the detailed statistical distribution information of volcano plots for a cleaner, more focused view of selected genes of interest. This approach allows researchers to easily track expression patterns of important genes across experimental conditions, enabling more efficient identification of consistent or divergent gene behaviors. Our example, shown in Fig. 1d, displays four key genes linked to neurodegeneration: SPP1, APOE, SERPINA1, and PINK1 across 5 disease comparisons: Frontotemporal Dementia (FTD) versus Control (CT), Multiple Sclerosis (MS) versus CT, Huntington’s Disease (HD) versus CT, Alzheimer’s disease (AD) versus CT, and Autism spectrum disorder (ASD) versus CT. The data was obtained from the ZEBRA database (Flotho *et al.* 2024). We further computed the differentially expressed genes split by sex using methods analogous to those reported in the database, highlighting another key feature of DominoPlots—the ability to display multiple comparisons across two distinct experimental setups. This visualization has also been used in a study by Graf *et al.* (2025) for comparing Parkinson’s disease-specific genes with Covid specific genes in two examined brain regions. For using the DominoPlot, we recommend discarding the dice background coloring to avoid overcrowding the plot, but the user is free to choose to ignore this recommendation.

## 4 Using DicePlots on maps

As a final addition, we implemented a routine supporting the dice layout on geographic maps to enable visualization on spatial data.

## 5 Conclusion

This note presents a user-friendly plotting library for visualizing multiple categorical dimensions in a single view. This effectively extends the repertoire of available visualizations by bridging the gap between high-level overviews and low-level detailed views, making it a valuable addition to existing tools. The examples demonstrate the core use cases, highlighting how the plots emphasize key data features in Dice- and DominoPlots. Although the tool enhances categorical visualization, it is currently limited by the number of features it can display per dimension. Therefore, careful selection of rows and columns is essential to maintain clarity. Future work focuses on improving compatibility with ggplot2 and introducing extensions such as ggdice and ggdomino.

## Data Availability

The code of the package is available at https://github.com/maflot/DicePlot, on CRAN (R Core Team 2013) https://CRAN.R-project.org/package=diceplot (R implementation), and https://github.com/maflot/pyDicePlot/ (Python implementation). The Python package is also available as pydiceplot package on pip. Use-case scenarios and additional documentation can be found at https://dice-and-domino-plot.readthedocs.io/en/latest/
